# Wheat Antipodal Cells with Polytene Chromosomes in the Embryo Sac Are Key to Understanding the Formation of Grain in Cereals

**DOI:** 10.3390/biology11091340

**Published:** 2022-09-11

**Authors:** Tatiana V. Doronina, Vasily V. Ashapkin, Elena M. Lazareva

**Affiliations:** 1 Biology Faculty, Lomonosov Moscow State University, Moscow 119234, Russia; 2 Belozersky Institute of Physico-Chemical Biology, Lomonosov Moscow State University, Moscow 119234, Russia; 3 All-Russia Research Institute for Agricultural Biotechnology, Moscow 119234, Russia

**Keywords:** wheat, embryo sac, antipodal cells, polytene chromosomes, differentiation, programmed cell death

## Abstract

**Simple Summary:**

The present work is devoted to the features of antipodal cells at the early stages of wheat seed development, ensuring the formation and protection of a full-fledged grain. Using the methods of cell and molecular biology, changes in the structure of these cells and their components during active functioning and death are shown. For the first time, data on the expression of some genes confirming the functions of antipodal cells were obtained. The characteristic features of cells at the stages of death were revealed. The data obtained indicate the key role of antipodal cells in the process of grain maturation and subsequently allow us to identify substances that ensure the formation of tissue, which is the food of the whole world.

**Abstract:**

The ultrastructure of antipodal cells of the *Triticum aestivum* embryo sac was studied at different stages of differentiation and programmed cell death. The importance of cell function in the antipodal complex is evidenced by the fact that it is fully formed before double fertilization, past the stages of proliferation of three initial cells, and several rounds of genome endoreduplication during differentiation. In this study, we showed that the actively synthesizing organelles, the granular reticulum, and Golgi apparatus, alter their structure during differentiation and death. The polymorphism of the shape of the mitochondria and plastids was demonstrated. For the first time, the actin filaments of the cytoskeleton and numerous multivesicular bodies associated with the plasma membrane were detected in the cytoplasm. The transfer of cytoplasm and organelles between antipodal cells and into the coenocyte of the endosperm was confirmed. DNA breaks and the release of cytochrome c at various stages of death were revealed. To understand the function of the antipodal cells, a quantitative PCR analysis of the expression of wheat genes involved in protective, antistress, and metabolic processes was carried out. We found that gene expression in the antipodal cell fraction was increased compared with that in the whole embryo sac. On the basis of the data, we assume that antipodal cells produce both nutrients and numerous antistress factors that ensure the normal development of the endosperm of the grain, which, in turn, further ensures the development of the embryo.

## 1. Introduction

The heart of the ovule of cultivated cereals is the embryo sac. Before double fertilization and after the proliferation of three initial antipodal cells and several rounds of endoreduplication of their genomes (up to 4c), a three-layer complex of haploid cells with giant polytene chromosomes is formed in the chalazal region of the embryo sac. The antipodal complex includes three cell layers: a basal layer adjacent to the nucellus, an apical layer bordering the endosperm, and a middle layer in between. Following fertilization, further rounds of endoreduplication occur and giant polytene chromosomes (maximum ploidy is 250c) are formed [1,2]. The nuclei of the cells of the various antipodal complex layers undergo a different number of rounds of endoreduplication [3].

Double fertilization leads to the formation of a noncellular endosperm and embryo. The nuclei of the endosperm divide synchronously, forming a coenocyte that borders the apical layer of a complex of highly polyploid antipodal cells. Wheat–rye hybrids exhibit anomalies of the antipodal complex that cause the disruption of the endosperm, and “unfulfilled” weak grain is formed [4,5]. Therefore, it is difficult to overestimate the role of antipodal cells in the development of the endosperm of cultivated cereals, in which the endosperm occupies the maximum volume of the mature grain. The importance of the formation of the antipodal complex is evidenced by the fact that even in the absence of double fertilization, the antipodal complex is formed completely, and actively transcribing polytene chromosomes are detected in the cells [1].

In most modern studies, however, the structure, function, and molecular processes that participate in the various stages of the ontogenesis of antipodal cells have been studied superficially or fragmentally. Therefore, for researchers of the female gametophyte of cultivated cereals, the most recognized cells with giant chromosomes are, according to Engel’s apt expression, “stepchildren” [6]. Perhaps this is because the cells of the antipodal complex are difficult to separate from the endosperm to obtain enough material for molecular studies. The study of the ultrastructure of antipodal cells requires experience in the total preparation of embryo sacs isolated from ovules. Moreover, the structure and possible function of antipodal cells change during differentiation and programmed cell death (PCD).

The literature contains many studies describing the cell structure of the antipodal complexes of the embryo sacs of cultivated cereals, but only at specific stages of development [1,4,6,7,8,9,10,11,12,13,14,15,16,17,18,19,20,21,22,23]. The main events, which include increasing the size and ploidy of the nuclei, changing the size and shape of the nucleoli, and changing the structure of the endoplasmic reticulum and mitochondria, that occur during the differentiation have been presented [2]. Structural features such as the convergence and unification of chromosomes, segregation of nucleolar components, nuclear envelope rupture, extrusion of the nucleolar components and chromatin, and DNA breaks that accompany the changes in the nuclei during PCD have been described in detail [3].

In the present study, we systematized the data, performed a comparative study of the features of the structure of the antipodal cells of the fertilized embryo sacs of *Triticum aestivum* at different stages of differentiation and PCD, and measured the gene expression in the antipodal polyploid nuclei.

## 2. Materials and Methods

The interphase haploid antipodal cells (n = 21) of the embryo sac of common wheat (*Triticum aestivum* L.) of the five varieties (Moscovskaya 35, Moscovskaya 39, Chinese Spring, Saratovskaya 29, Timiryazevskaya Jubilee; 2n = 42) were examined. We examined 500 embryo sacs (approximately 12,500 cells) for each variety.

Embryo sacs including the endosperm coenocyte (3n), embryo (2n), and antipodal cells with polytene chromosomes (n) were isolated from ovule tissues based on Petrova’s method [24].

We fixed the ovules in 4% paraformaldehyde (Cat no. 158127, Sigma, St. Louis, MI, USA) in PHEM buffer (pH = 6.9) for 2 h for the immunodetection of actin and tubulin and in 4% paraformaldehyde (Cat no. 158127, Sigma, St. Louis, MI, USA) in PBS (Cat no. 2810305, MP Biomedicals, LLC, Illkirch, France; pH = 7.2) for 2 h for the immunodetection of cytochrome c. We fixed the ovules in 0.1 M Sorensen phosphate buffer (KH_2_PO_4_/Na_2_HPO_4_) (pH = 7.3) containing 2.5% glutaraldehyde and sucrose (0.015 g/mL) for electron microscopy (SKU 02198595-CF; MP Biomedicals, LLC, Illkirch, France) for 2 h.

To study the structure of the antipodal cell nuclei, DNA was stained with DAPI fluorochrome (CAS Number: 28718-90-3, Sigma, St. Louis, MI, USA).

To detect DNA strand breaks by a terminal deoxynucleotidyl transferase dUTP nick end labeling (TUNEL) assay, we used the TUNEL Assay Kit (CAS Number: K3000, Sileks, Moscow, Russia).

Rabbit polyclonal antibodies (1:100 dilution in PBS + 0.1% BSA) against cytochrome c (AS08 343A, Agrisera, Vännäs, Sweden) were used for the indirect immunocytochemical detection of mitochondria. Donkey antibodies (1:1000 dilution in PBS + 0.1% BSA) against rabbit IgG conjugated with fluorochrome Alexa 488 (ab150073, Abcam, Cambridge, UK) were used as the secondary antibodies. For the indirect immunocytochemical detection of microtubules and actin filaments, mouse monoclonal antibodies (1:250 dilution in 10 mM TRIS buffer (pH = 7.6) + 0.1% BSA) against α-tubulin clone DM1a (SKU 05-829, Sigma, St. Louis, MI, USA) and mouse monoclonal antibodies (1:100 dilution in PBS + 0.1% BSA) against the actin clone 10-B3 (MabGPa) (A 0480, Sigma, St. Louis, MI, USA) were used. Donkey antibodies (1:1000 dilution in PBS + 0.1% BSA) against mouse IgG conjugated with fluorochrome Alexa 488 (ab150109, Abcam, Cambridge, UK) were used as the second antibodies for actin detection and donkey antibodies (1:1000 dilution in 20 mM TRIS buffer (pH = 8.2) + 0.1% BSA) against mouse IgG conjugated with fluorochrome Alexa 488 (ab150109, Abcam, Cambridge, UK) were used as the second antibodies for the detection of microtubules. Preparations were incubated with primary antibodies overnight, RT, with secondary antibodies for 1 h, 37 °C. 

Periodization of cell development in the antipodal complexes was performed to correlate the morphology of antipodal cells with time after pollination [25]. Plants that had just emerged from the leaf sheath and still contained green anthers and unshelled stigmas were selected as mother plants. In preparation for castration, the upper and lower poorly developed spikelets were removed from the mother wheat plants. The central flowers were removed from the remaining spikelets using tweezers, and only two lateral flowers were left. The awns and upper parts of the glumas were removed. During the castration procedure, the stamens of the flowers were removed using tweezers, leaving only the pistils. The mother plants were placed under insulators. Limited free pollination was used for hybridization. For this, the ears of the paternal form were selected, in which one or two flowers had begun to bloom. These were cut off, and the awns and upper portion of the spikelet scales were removed. Two paternal and one maternal plants were placed under each hybridization insulator. The following time points for fixation and study were used: 0, 1, 2, 3, 4, 5, 6, 7, 8, 9, and 10 days after pollination.

The preparations were examined using a Leica light microscope and Axiovert 200M fluorescence microscope (Carl Zeiss Inc., Oberkochen, Germany). 

Electron microscopy was performed according to a standard method as described previously [3].

Image processing was performed using ImageJ and Photoshop CS6 software.

To obtain the RNA, samples of the antipodal cells, embryo sacs, and roots were fixed in RNAlater preservation solution (Qiagen, Hilden, Germany). To isolate the RNA, the fixing solution was removed, a lysis buffer containing β-mercaptoethanol was added to the samples from the Spectrum Plant Total RNA Kit (Sigma-Aldrich, St. Louis, MI, USA), and the tissues were homogenized with a Teflon pestle. Additional steps were carried out according to the manufacturer’s instructions. First strand cDNA was synthesized using the MMLV RT Kit (Eurogene, Moscow, Russia). Equal aliquots of the reaction mixtures were used directly for quantitative PCR using qPCRmix-HS SYBR Kits (Eurogene, Moscow, Russia) and a LineGene 9660 amplifier (Bioer Technology, Hangzhou, China) using the following program: 95 °C for 5 min (matrix denaturation and DNA polymerase activation), followed by 40 cycles of 94 °C for 15 s (denaturation) and 60 °C for 1 min (annealing/elongation). Gene expression was determined relative to the reference gene *Ta54227* [26] and calculated using the ΔCq method. Three independent expression measurements were taken for each gene. The statistical processing of the results was performed using Microsoft Excel.

## 3. Results

We considered the structural features of the antipodal cells with polytene chromosomes during the last two stages of ontogenesis. At different stages of differentiation and PCD in the wheat embryo sac of antipodal cells, we identified their early, middle, and late stages based on morphological features [2,3].

We established a correlation of the characteristic morphological features of the antipodal cells during differentiation and PCD at specific times after the pollination of the mother plant. We found that early differentiation of antipodal cells occurred 24 h after pollination, the middle stage after 48 h, and the late stage after 72–96 h. The early stage of cell death was detected after 120 h (5 days), the middle after 154–178 h (6–7 days), and the late stage (complete absorption of cells) 8–10 days after pollination.

**Figure 1:**
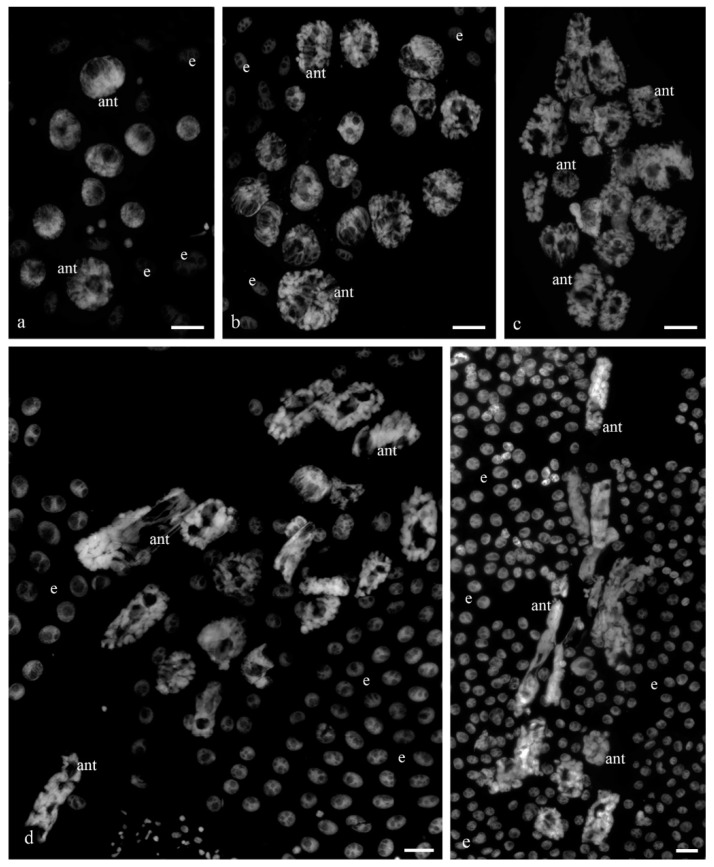
A general view of the antipodal complexes of the wheat embryo sacs at various stages of differentiation and programmed cell death. DAPI. Stages: early differentiation—(**a**); middle differentiation—(**b**); late differentiation—(**c**); stage of early PCD—(**d**); middle PCD—(**e**). ant—antipodal nuclei; e—endosperm nuclei. The scale is 30 µm.

**Figure 2:**
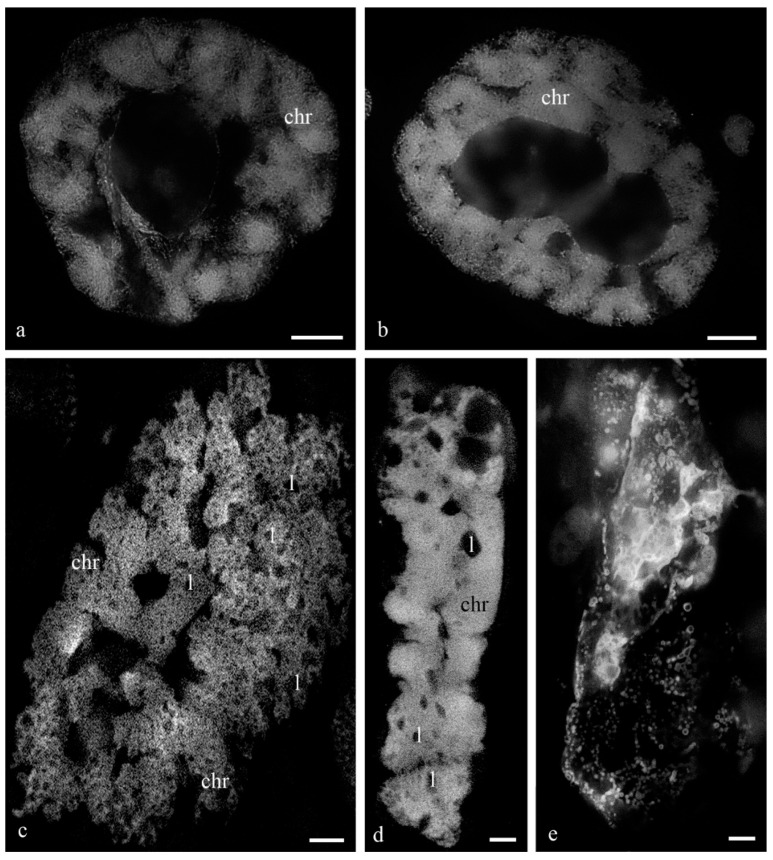
The nuclei of the antipodal cells of the wheat embryo sacs with giant polytene chromosomes at the stages of differentiation and programmed cell death. DAPI. The antipodal nucleus at the stage of middle differentiation, individual fibrils—chromatids are visible in the bodies of the polytene chromosomes (**a**); the nucleus of the antipodal cell at the late stage of differentiation, dense bodies of polytene chromosomes are visible (**b**); the nucleus at the middle stage of PCD, merged and united cellular polytene chromosomes (**c**); the nucleus at the late stage of PCD, dense united polytene chromosomes of the nucleus (**d**); fragmented nucleus at the late stage of PCD (**e**). chr—polytene chromosomes; l—lacunae in the region of polytene chromosomes. The scale is 10 µm.

Figure 1 shows the antipodal complexes of wheat embryo sacs at the early, middle, and late stages of differentiation (Figure 1a–c) and the middle and late stages of PCD (Figure 1d,e). It is evident that during differentiation, round nuclei with a diameter of up to 20–30 µm (Figure 2a) increased in size, and later, the nuclei adopted an oval shape of up to 30 × 80 µm (Figure 2b). At the stage of death, the nuclei were stretched (up to 100 microns long and 10 microns wide). At the stage of differentiation, individual polytene chromosomes and 1–2 nucleoli were observed in the antipodal nuclei (Figure 2a,b). At the various stages of death, the chromosomal regions converged, united, and subsequently fragmented (Figure 2c–e).

**Figure 3:**
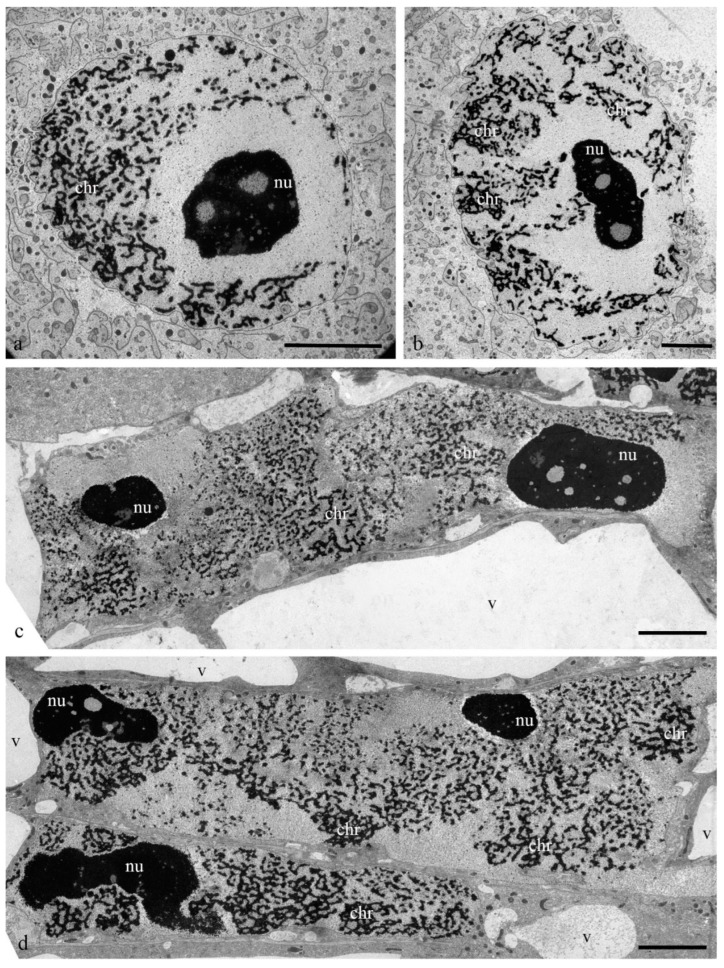
Ultrastructure of the nuclei of the antipodal cells of wheat embryo sacs at various stages of differentiation. Round nuclei of antipodal cells at the early stage of differentiation (**a**,**b**); elongated nuclei of antipodal cells at the middle (**c**) and late stages of differentiation (**d**). chr—polytene chromosomes; nu—nucleolus; v—vacuoles. The scale is 10 µm.

During differentiation, the ultrastructure of the main components of the antipodal nuclei changed (Figure 3). At early stages, chromatids (0.3–0.4 µm thick) were observed in rounded nuclei as a part of individual polytene chromosomes (Figure 3a,b) and large nucleoli. Separate transcription sites were observed. Later, the nuclei elongated, the nucleoli lost their oval shape (Figure 3c,d), and the polytene chromosomes converged.

**Figure 4:**
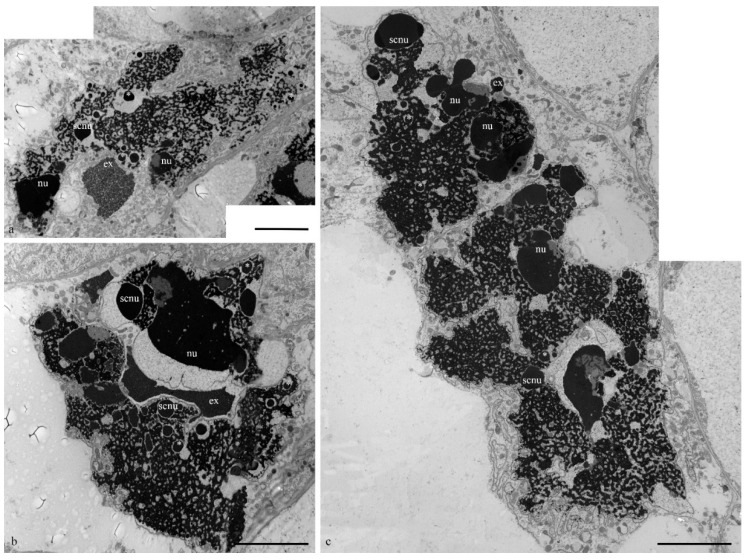
Ultrastructure of the wheat embryo sac nuclei at the middle and late stages of programmed cell death. Segregated nucleolus components in the lacunae of polytene chromosomes of antipodal nuclei (**a**–**c**) and nucleolus extrusion (ex). nu—nucleolus; scnu—large segregated nucleolus components, *—small segregated nucleolus components. The scale is 10 µm.

**Figure 5:**
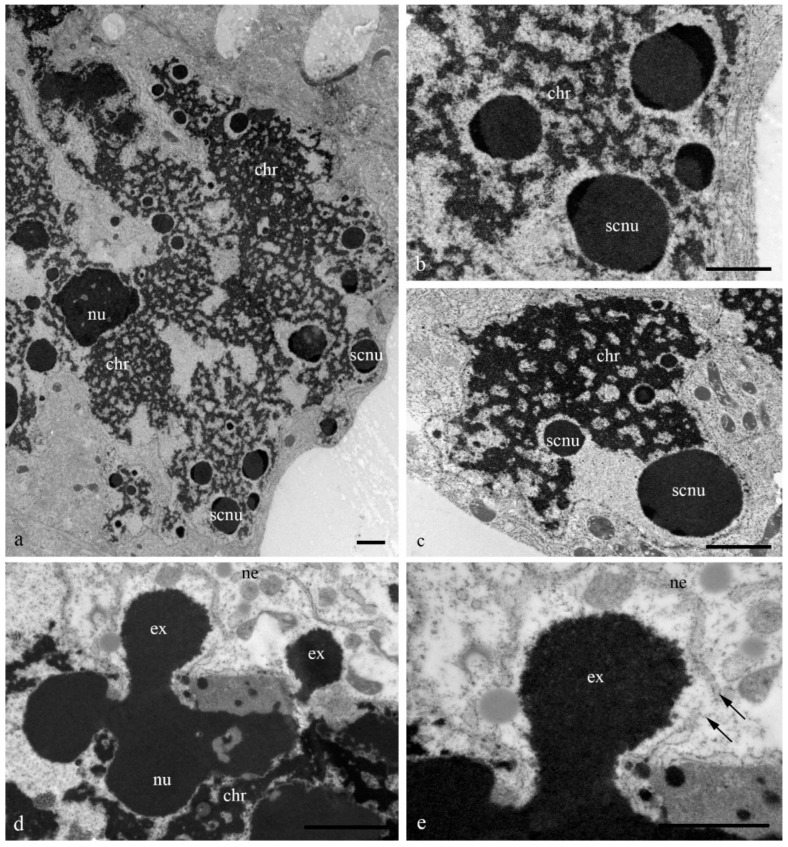
Ultrastructure of the wheat embryo sac nuclei at the middle and late stages of programmed cell death. Segregated nucleolus components in the lacunae of polytene chromosomes (chr) of antipodal nuclei (**a**–**c**) and nucleolus extrusion (ex) through nuclear envelope breaks (**d**,**e**; arrows). nu—nucleolus; scnu—segregated nucleolus components, ne—nuclear envelope. The scale is 2 µm.

During the death stages of antipodal cells, the ultrastructure of the nuclei changed (Figure 4). The nucleoli acquired an unusual shape, in which some of the components were detected on the surface of the chromatids and the extrusion of the granular component was present in the cytoplasm. Numerous extended invaginations of the nuclear envelope occurred, and numerous segregates of the nucleolar components with different shapes and sizes (Figure 4; Figure 5a–c) and numerous RNA transcripts were evident in the lacunae of the polytene chromosomes. The integrity of the nuclear envelope at the extrusion sites of the nucleus and nucleolus material was disrupted (Figure 5d,e).

**Figure 6:**
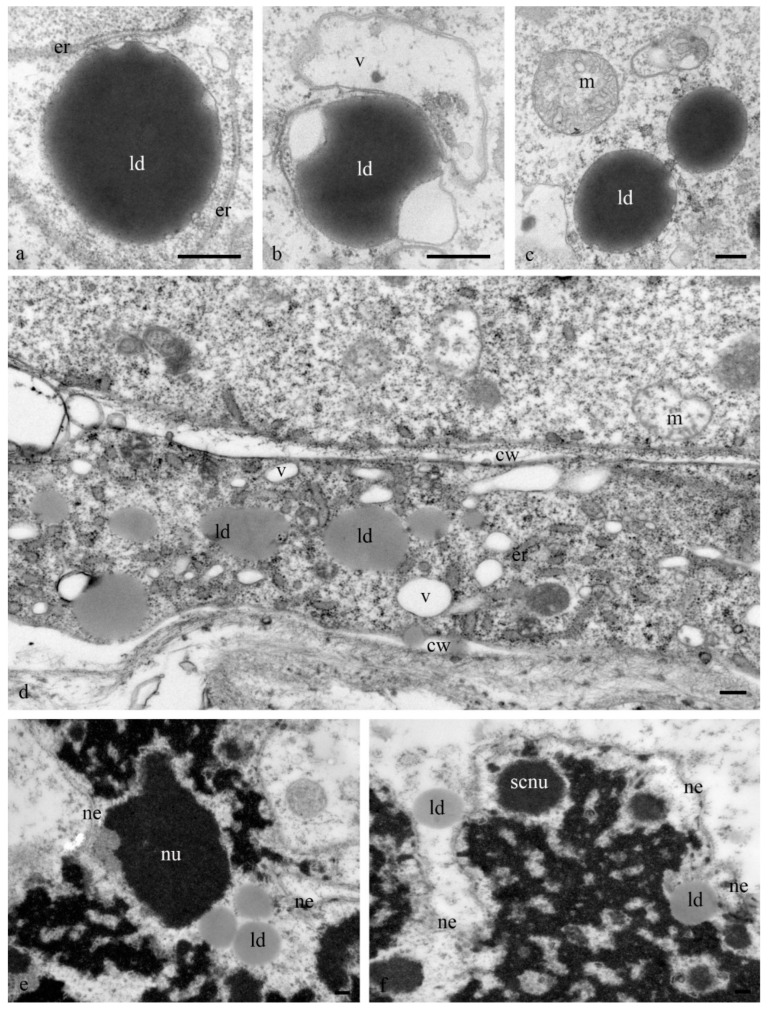
Ultrastructure of the lipid droplets of the antipodal wheat embryo sac cells at various stages of differentiation and programmed cell death. Lipid droplets (ld) in the cytoplasm at the early stage of differentiation (**a**–**d**) and in the nuclei during PCD (**e**,**f**). ld—lipid droplets; cw—cell wall; ne—nuclear envelope; nu—nucleolus; v—vacuoles; m—mitochondria; chr—polytene chromosomes; scnu—segregated components of the nucleolus; er—granular reticulum. The scale is 2 µm.

Numerous lipid droplets were detected in the cytoplasm of antipodal cells at the early and middle stages of differentiation near the cisterns of the granular endoplasmic reticulum and mitochondria (Figure 6a–d). During the late stages of death, lipid droplets were observed in the nucleus (Figure 6e,f).

**Figure 7:**
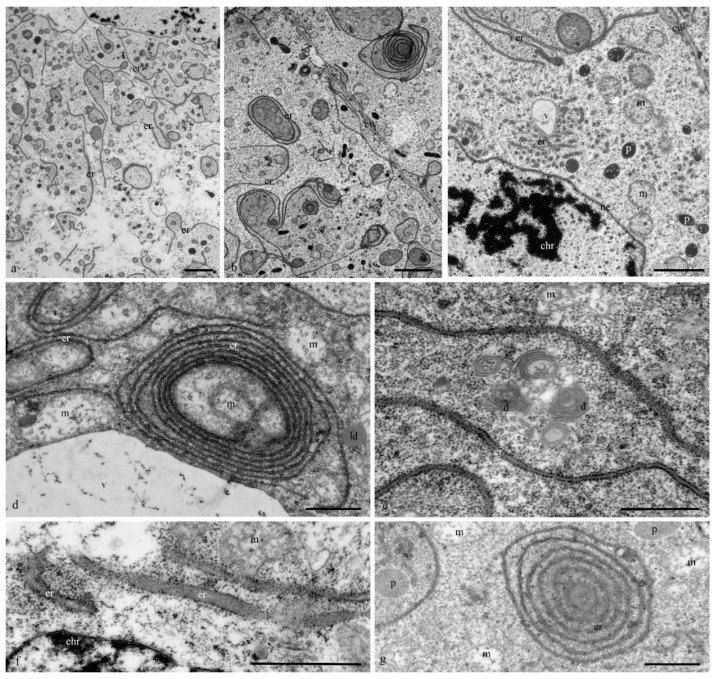
Ultrastructure of the granular reticulum of the antipodal cytoplasm of the wheat embryo sac cells. A network of extended flat cisternae (**a**–**c**) and tubes (**b**) of granular reticulum at an early stage of differentiation. Concentric circles (**d**,**g**) and expanded cisternae of the granular reticulum during PCD. Ne –nuclear envelope; chr—polytene chromosomes; er—granular reticulum; m—mitochondria; p—plastids; d—dictyosomes; v—vacuoles; ld—lipid droplets; cw—cell wall. Scale: (**a**–**c**)—10 µm; (**d**–**g**)—5 µm.

The structure of the granular reticulum at the early stages of differentiation appeared as an extensive network of flat cisternae (20 nm) (Figure 7a,b,e) near the nucleus. Separate areas of the cell cytoplasm were occupied by numerous tubules (diameter, 40 nm) covered with ribosomes (Figure 7c). Later and during cell death in the cytoplasm of the antipodal cells, concentric circles of granular reticulum (Figure 7d,g) and expanded cisternae (more than 60 nm) were observed (Figure 7f).

**Figure 8:**
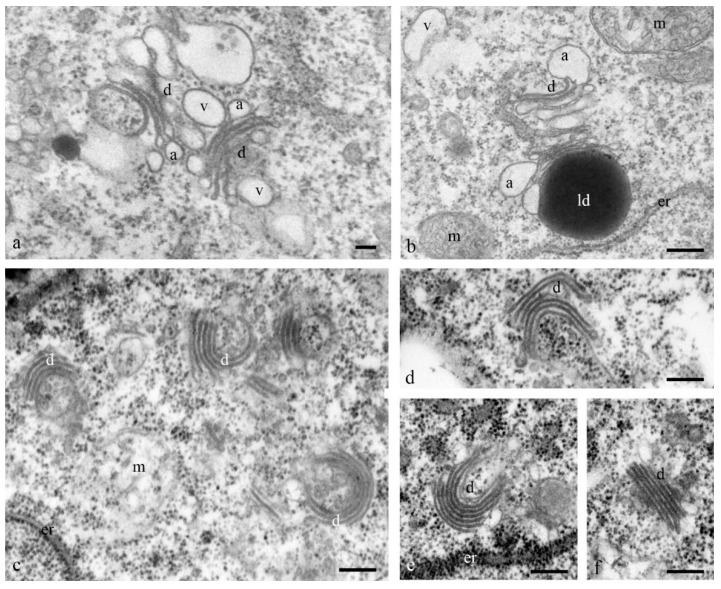
Ultrastructure of the Golgi apparatus in the cytoplasm of the antipodal cells. At the early stage of differentiation, individual dictyosomes consist of 5–7 flat cisternae with expanded ampoules (**a**,**b**). During programmed cell death, the stacks of dictyosome cisterns are bent (**c**–**f**). d—dictyosomes. a—ampoules of cisternae, er—granular reticulum; m—mitochondria; v—vacuoles. The scale is 100 nm.

At the early stages of the differentiation of antipodal cells, the dictyosomes of the Golgi apparatus exhibited expanded ampoules at the ends of each of the 6–7 cisternae of the stack (Figure 8a,b). During later stages of complex development, the ordered (parallel) stacks of cisternae of most dictyosomes were strongly bent and the cisternae of the Golgi apparatus had different lengths (Figure 8c–f).

**Figure 9:**
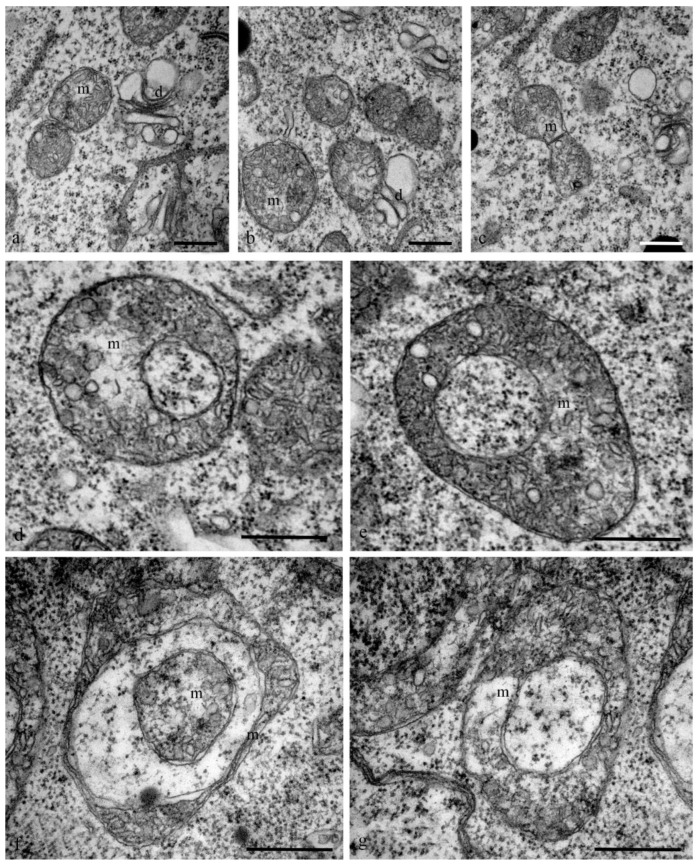
Ultrastructure of the mitochondria of the cytoplasm of the antipodal cells of the wheat embryo sac. All mitochondria of the antipodal cells have finger-shaped, disordered cristae. At the early stages of differentiation, mitochondria are round with a diameter from 0.5 to 1 µm (**a**–**c**), cup-shaped mitochondria with a diameter of 1 µm (**d**,**e**) are evident, and cup-shaped mitochondria (**f**,**g**) are more often detected during PCD. m—mitochondria; d—dictyosomes. The scale is 500 nm.

Mitochondria also exhibited changes in their structure. At the early stages of differentiation, the mitochondria were small rounded organelles (diameter, 200 nm) with finger-shaped crystae (diameter, 10–100 nm) (Figure 9a–c). At later stages, cup-shaped mitochondria (diameter, 1.5 microns) were more frequently observed in the cells (Figure 9d–g).

**Figure 10:**
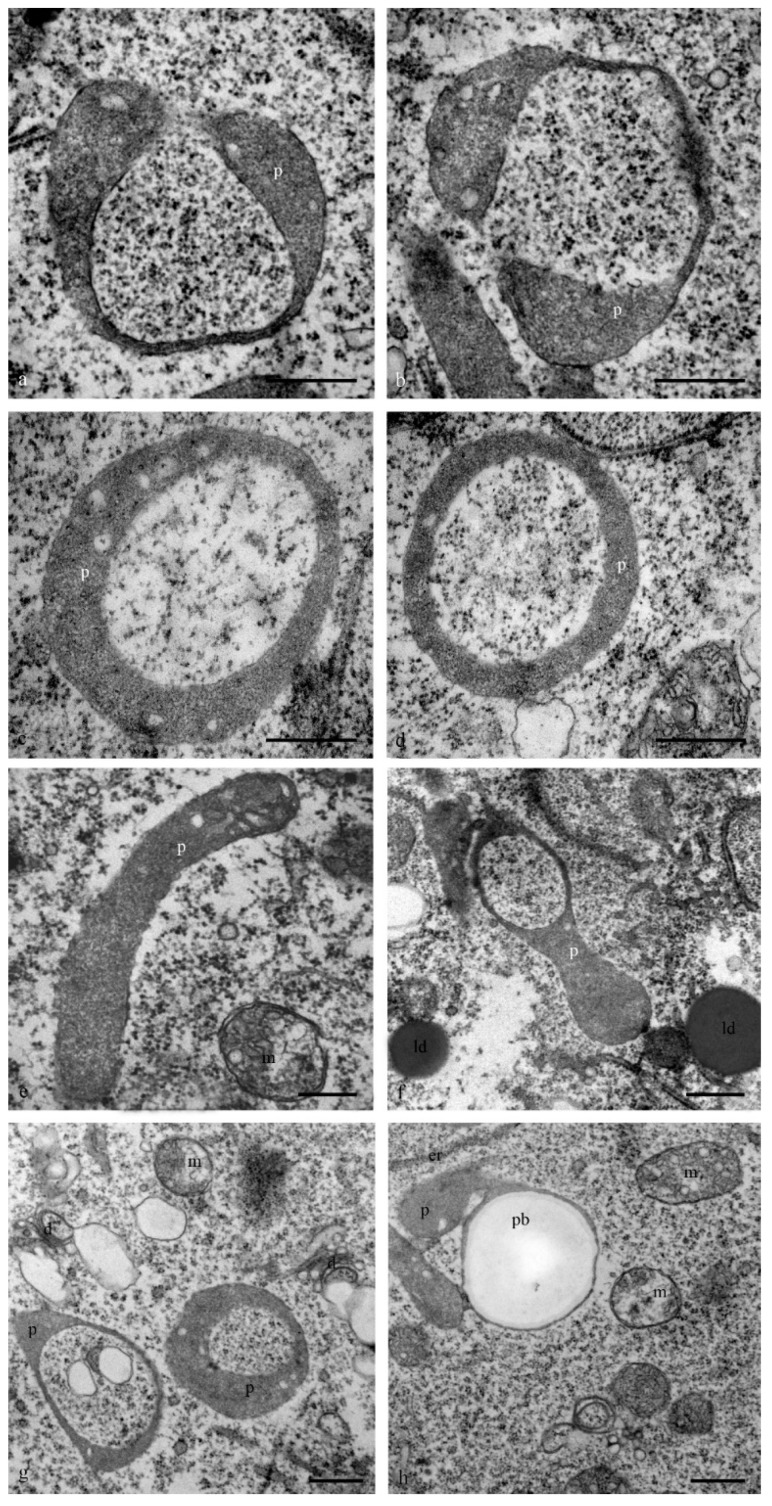
Ultrastructure of the plastids (leukoplasts) of the cytoplasm of the antipodal cells. At stages of active cell functioning, the shape and size of the plastids vary. A granular stroma, and single lamellae and several small starch grains (**a**–**g**) are evident. Plastids can have constrictions (**a**,**b**), a cup shape (**c**,**d**,**g**), a stick shape, a cup-shaped area (**e**,**f**), or a complex shape (**h**). p—plastids; m—mitochondria; d—dictyosomes; pb—protein body; ld—lipid droplets. The scale is 500 nm.

At the early stages of complex development, the leucoplasts (plastids) of antipodal cells acquired a diverse shape: ring-shaped with constrictions and cup-shaped areas were evident (Figure 10). Several lamellae, starch grains, and later, small plastids with a dark stroma and a small number of starch grains, were observed.

**Figure 11:**
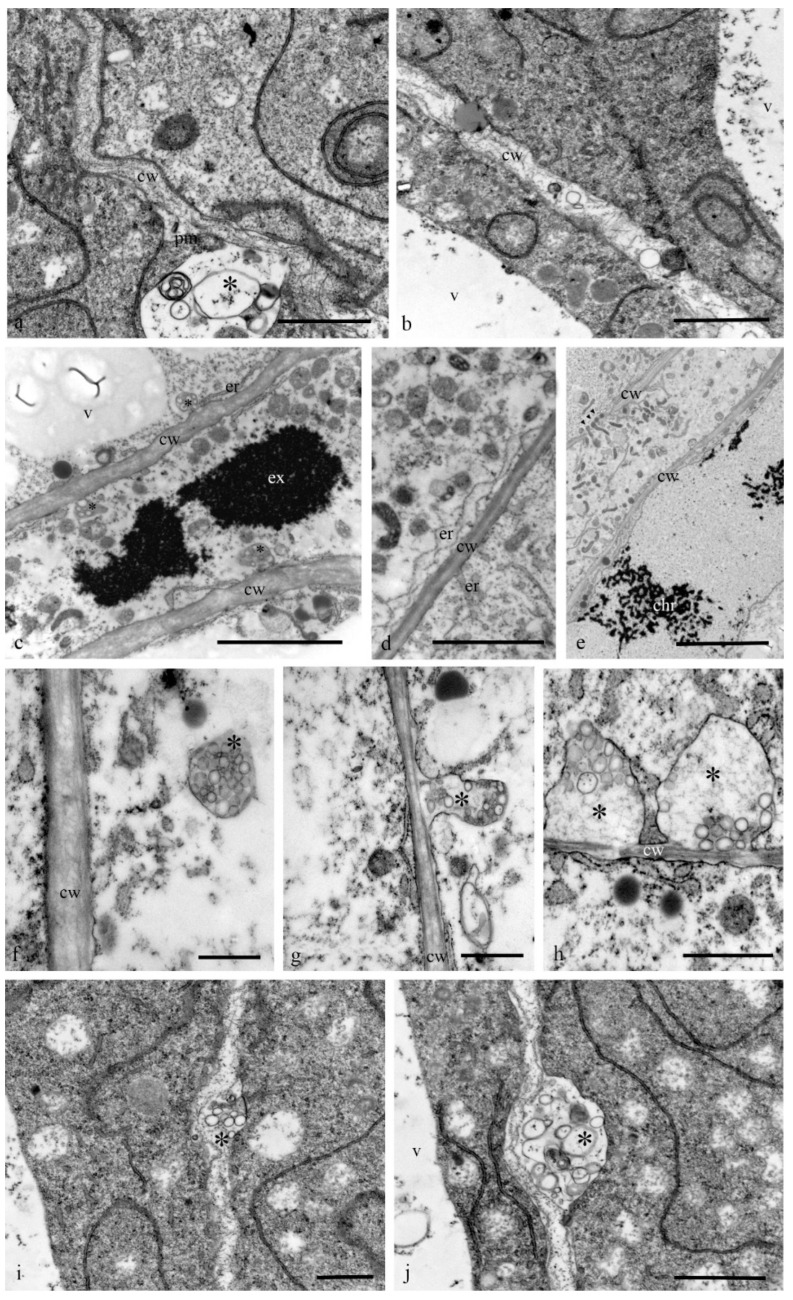
Ultrastructure of the cell walls and multivesicular bodies of the antipodal cells of the wheat embryo sac. The cell walls between antipodal cells have an unequal thickness and disordered hemicellulose microfibrils (**a**,**b**). Expanded cisternae of the granular reticulum may be observed near the plasma membrane (**c**), and the cavities of the reticulum cisternae are often fused with the plasma membrane (**d**). The transfer of various organelles from cell to cell through wide channels in the cell wall can be observed (**e**). In the cytoplasm of antipodal cells, exocytosis or endocytosis of the multivesicular bodies is evident, often combined with the plasma membrane (**f**–**j**). Multivesicular bodies (*) were found in the area of the cell walls. cw—cell wall; pm—plasma membrane; v—vacuoles; er—granular reticulum; ex—granular reticulum; chr—polytene chromosomes. Scale: (**a**)—2 µm; (**b**–**d**)—5 µm, (**e**)—10 µm, (**f**)—2 µm; (**g**,**h**)—1 µm, (**i**,**j**)—2 µm.

The structure of the cell walls between the antipodal cells was heterogeneous. The thickness changed, and the cell wall contained disordered hemicellulose microfibrils. Exocytosis or endocytosis of multivesicular bodies within the cell walls or apoplast was frequently observed (Figure 11). The transfer of organelles including plastids and mitochondria through wide channels was observed in the cell wall between the cells (Figure 11e) and between the antipodal cells and coenocyte of the endosperm.

**Figure 12:**
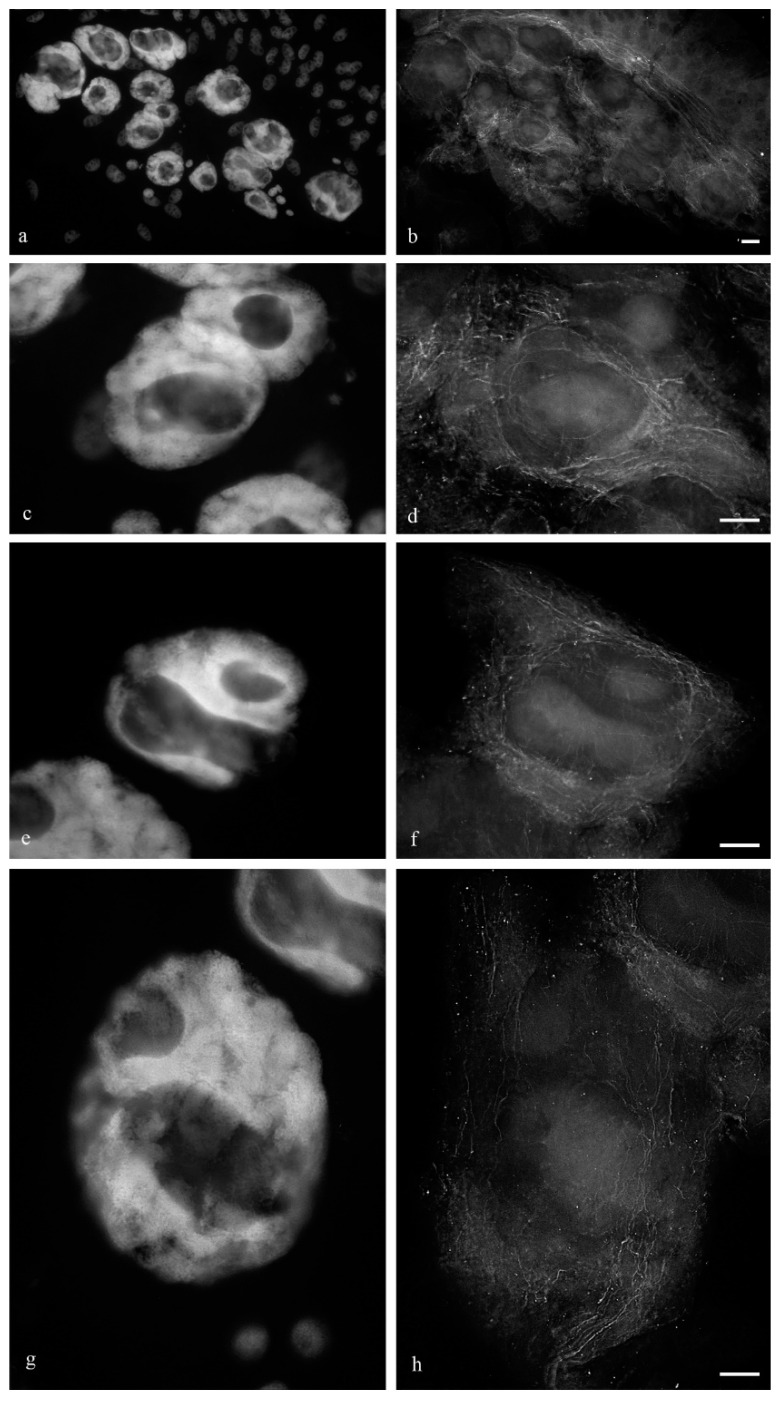
Actin filaments of the cytoskeleton of the cytoplasm of the antipodal cells of the wheat embryo sac at the early and medium stages of differentiation. Microfilaments surround the nuclei of the antipodal cells of the complex (**a**) and are detected between vacuoles and on the periphery of the cytoplasm (**b**). Microfilaments of the individual antipodal cells of the complex (**c**,**e**,**g**—DAPI); actin filaments (**d**,**f**,**h**). The scale is 10 µm.

**Figure 13:**
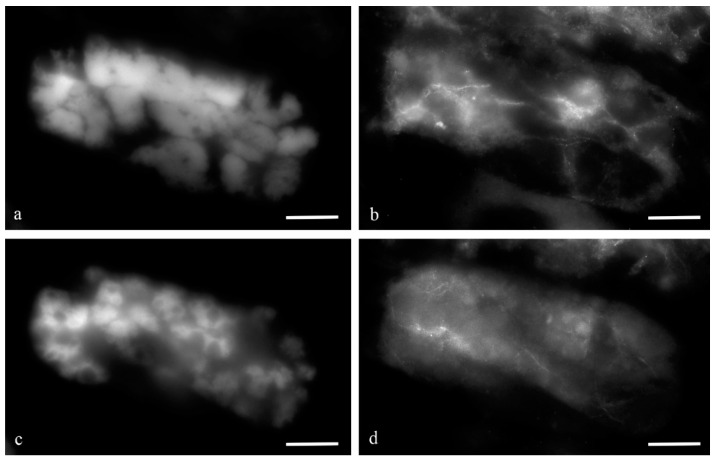
Bundles of microtubules of the cytoskeleton of the cytoplasm of the antipodal cells of the wheat embryo sac at the middle stage of programmed cell death. The structure of the chromosomes of the nucleus of the antipodal cell is represented in two optical slices (**a**,**c**, DAPI). Rare, fragmented bundles of microtubules are concentrated in the cytoplasm with the invaginations of the nuclear envelope around polytene chromosomes (**b**,**d**, DM1a).

During the middle stage of PCD, a branched network of actin filaments (Figure 12) and thin network of disordered and fragmented microtubule bundles (Figure 13) were observed. During the death of antipodal cells, the microtubule network was disassembled and bundles of actin filaments were thinned.

**Figure 14:**
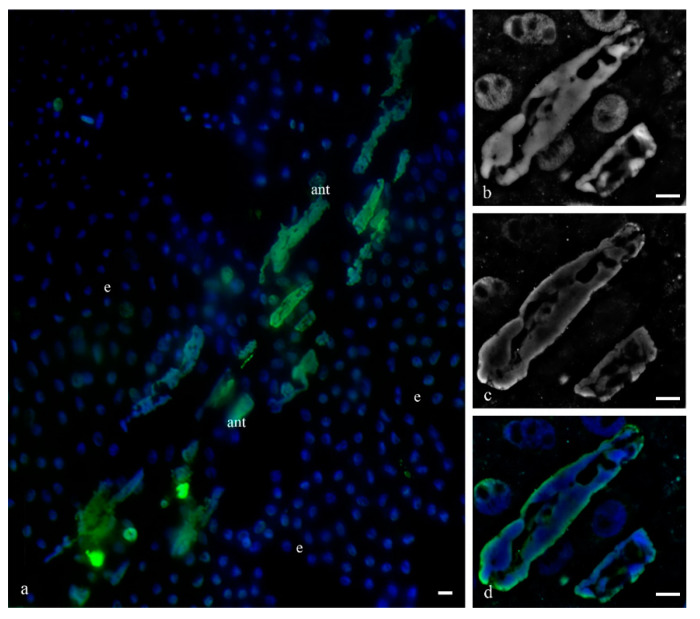
DNA breaks in the nuclei of the wheat antipodal cells detected by the TUNEL assay at various stages of PCD. Breaks were not detected in all cells of the antipodal complex (**a**), and most of the breaks were detected in DNA at the periphery of the nucleus (**b**–**d**) or were detected in the entire volume of the chromosomes (**a**). ant—antipodal nuclei, e—endosperm nuclei. The scale is 10 µm.

**Figure 15:**
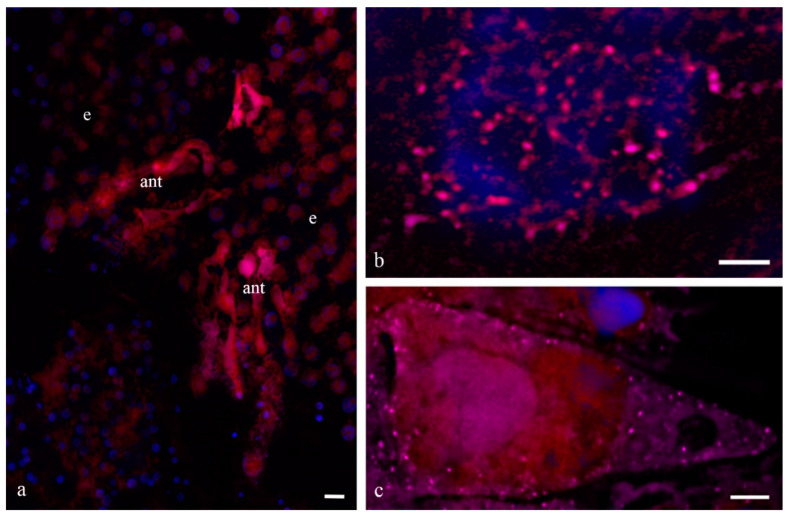
Localization of cytochrome c in the cytoplasm of the wheat antipodal complex cells during PCD. A complex of antipodal cells (**a**). Cytochrome c in the mitochondria of a functioning antipodal cell (**b**) and in the cytoplasm of the antipodal cell during PCD (**c**). ant—antipodal, e—endosperm. Scale 20 µm.

To identify the manner of cell death of the antipodal complex, DNA breaks in the nuclei were detected (Figure 14) as well as the immunocytochemical detection of cytochrome c (Figure 15). We detected breaks in the DNA of most nuclei of the cell complexes at various stages of PCD. The localization of ruptures in different nuclei differed. Ruptures were detected on the periphery of the nucleus or occupied the entire volume of the nuclei. During cell death, cytochrome c was released in the cytoplasm of almost all cells of the antipodal complex (Figure 15).

To study the gene expression levels, we selected *YUCCA 9* (auxin metabolism), *AT3G12580* (cytochrome p450), *AT5G01860* (C2H2 and C2HC zinc finger superfamily protein), *At1g77790* (glycosyl hydrolase family 17 protein), *At3g14630* (Hsp70), and *At2g31030* (oxysterol-binding family protein). Orthologs for these genes in wheat were identified using the Plaza database (Table 1). Primers for these genes are given in Supplementary Table S1. Relative expression levels are shown in Table 2. For comparison, we determined the relative expression of the same genes in the cells of the whole embryo sac and root (Table 3 and Table 4). The genes of cytochrome p450 (4.6-fold higher than the reference gene) and Hsp70 (10.4-fold higher than the reference gene) were highly expressed in the antipodal cells. The comparison of the data of the pure fraction of antipodal cells with the fraction of all embryo sac cells (containing the embryo, endosperm, and antipodal cells) indicated that the expression of all genes, with the exception of *YUCCA 9*, in the antipodal cells was greater than that in the embryo sac cells. Cytochrome p450 gene expression was 1.9-fold higher, the C_2_H_2_ and C_2_HC zinc finger superfamily protein was 2.1-fold higher, the glycosyl hydrolase family 17 protein was 3.9-fold higher, Hsp70 was 9.5-fold higher, and the oxysterol-binding family protein was 6.3-fold higher. It should be noted, however, that the expression of most of the genes studied was higher in the root cells compared with the antipodal cells and embryo sac cells. The only notable exception was the Hsp70 gene, which was 1.8 higher in the antipodal cells compared with the root cells.

## 4. Discussion

The antipodal cell complex is localized in the chalazal part of the embryo sac of plants. Antipodal cells of different plant species are characterized by a variability in their morphology, number, and lifespan [27]. Antipodal cells in most plants die before or shortly after fertilization. However, in some plants, particularly cereals, antipodal cells persist for a long time after fertilization. During proliferation, three initial antipodal cells divide to form three layers. Giant polytene chromosomes are formed in the nuclei after several rounds of endoreduplication [1].

In *Zea mays*, 20 [14] to 25 [28] antipodal cells were detected. In *Orysa sativa*, different groups observed 3–5, 5, 5–10, 10–15, and 6–20 antipodal cells, whose lifespan lasted three days after pollination [21]. Complexes of antipodal cells from different *Triticale* varieties were analyzed, and the life expectancy varied from 96 to 120 h after pollination [15]. In *Hordeum vulgare*, 35–55 [6] and 50–70 [29] antipodal cells were detected with lifespans of 5–6 days after pollination. In *Secale cereale*, 16–32 antipodal cells were detected. In wheat, 8–20 [30], 15–25 [16], 20 or more [20], 25–27 [1], and 24–36 [31] antipodal cells were detected along with a lifespan of 5–7 days [16,30].

In the wheat embryo sacs, we found that the number of antipodal cells varied from 20 to 27 and their life expectancy after fertilization was approximately 10 days. The differentiation stage required 4 days, and the first signs of death began to appear 4–5 days after pollination.

The number, life span, and size of the antipodal cells depend not only on the species of plant but also on the variety and conditions in which the plant grows. In studies conducted on the number of antipodal cells of two-row and six-row barley [32], wheat, rye, and their hybrids [31] as well as two-row barley of different varieties [33], they were compared while growing in a greenhouse and in a field [34]. The authors expressed the idea that the number of antipodal cells may vary and the necessary level of physiological activity may be achieved by the ratio of a certain number of cells and nucleus and nucleolus size. During unfavorable environmental conditions such as low temperature or poor light, the optimum level of the activity of antipodal cells may be achieved by increasing the number of cells with a decrease in the cell volume or by increasing the cell volume with a decrease in number [34].

The antipodal cells of most cereals, particularly *Orysa sativa* [21], *Hordeum vulgare* [6], *Triticale* [15], *Secale cereale*, and *Triticum aestivum* [19], have a single nucleus. Antipodal cells from *Zea mays* are multinucleated [13], and these cells contain 5–6 nuclei [28]. *Avena sativa* [35] and *Phlum boehmeri* [36] have both mononuclear and binuclear antipodal cells as part of the same antipodal complex.

The antipodal cells of cereals are large [6,13,14,15,19,21,22]. In *Orysa sativa*, the nuclei have a diameter greater than 25 microns [21], and the nuclei are approximately 60 microns in *Hordeum vulgare* [29]. Very large nucleoli containing vacuoles have been detected in the nuclei [6,13,14,15,19,21,22].

The structure of the polytene chromosomes of antipodal nuclei differs from that of polytene chromosomes in animals through the absence of the somatic conjugation of chromatids and, consequently, an associated transverse striation and pattern of disks and interdisks [1,2,29,37,38,39,40]. During the period of differentiation of antipodal cells following several rounds of endoreduplication, the ploidy of antipodal cells increases significantly, and in wheat, it can reach a maximum of 256C [2,3,16].

On the basis of our observations, the morphology of wheat antipodal complex nuclei changed during development. Antipodal cells in the early and middle stages of differentiation exhibited one large nucleus and from one to four nucleoli. The nuclei had a rounded shape, and the diameter was up to 20 microns at the early stage and up to 30 microns at the middle stage of differentiation. At the early stage of differentiation, individual polytene chromosomes were not observed in the nuclei. As the size of the nuclei increased, which occurred at the middle stage of differentiation, chromosomal territories were observed. During the late stage of differentiation and death, the nuclei were elongated and were nearly 30 microns in width and 80–100 microns in length. The bodies of the polytene chromosomes were compacted and united. During differentiation and death, numerous transcripts were detected near the polytene chromosomes, and in many cells at the stage of death, the extrusion of the granular component of the nucleolus occurred and the nuclear envelope ruptured. Thus, cells were provided with the necessary matrices and ribosomes for protein translation.

All studies devoted to antipodal cells have demonstrated a large number of organelles in the cytoplasm. The presence of an extensive network of endoplasmic reticulum, numerous mitochondria, plastids, and dictyosomes of the Golgi apparatus have been documented.

The mitochondria of *Zea mays* were oval or rounded in a cross-sectional view [13]. In antipodal cells of *Orysa sativa*, 10–15% of the cytoplasm was occupied by mitochondria before fertilization. The size of the plastids and mitochondria of *Orysa sativa* was 1–2 microns in length and 0.4 microns in width [21]. In *Hordeum vulgare*, the mitochondria from the early stages of antipodal cell development were evenly distributed throughout the cytoplasm. The number of mitochondria in antipodal cells of *Hordeum vulgare* decreased 40–50 h after fertilization [6]. In *Triticum aestivum*, the mitochondria had different shapes including a cup shape [19].

The plastids of antipodal cells from *Zea mays* and *Orysa sativa* contained insignificant amounts of starch grains or did not contain starch at all [14,21]. The number of lamellae was not significant [22]. In *Hordeum vulgare*, plastids exhibited a dark matrix and were round, ovoid, or elongated, with rare thylakoid membranes. Starch grains were not deposited. Plastids were evenly distributed throughout the cytoplasm, and their number in the ontogenesis of antipodal cells decreased earlier compared with the number of mitochondria [6]. The authors noted a diversity in the shape of plastids and mitochondria. The similarity of the plastid and mitochondrial forms has been shown in both the cytoplasm of wheat antipodal cells [19] and barley [6]. Mitochondrial shape polymorphism was observed in the cytoplasm of the antipodal cells of *Triticum aestivum* [19], *Hordeum vulgare* [6], *Orysa sativa* [21,22], and *Zea mays* [13].

We observed changes in the shape of the mitochondria in the cytoplasm of the wheat antipodal cells. At the early stages of differentiation, small round (0.5 microns in diameter) and cup-shaped mitochondria with disordered finger-shaped crystals were observed, whereas oval mitochondria (0.8 microns) were evident at the late stages of differentiation. Plastids at all stages of differentiation and death had a dark granular matrix, and they were rounded (0.5 microns in diameter) and elongated (2 microns in length), often with constriction and a cup shape. The plastids contained a small amount of starch grains and from one to eight disordered lamellae.

The ER cisterns of the antipodal cells varied in length and shape. *Zea mays* had short and extended ER cisterns [14]. Short ER cisterns located near the nucleus and cell wall were evident in the cytoplasm of *Orysa sativa* before fertilization [21], whereas after fertilization, concentric ER circles were prominent [22]. Parallel cisterns also prevailed in *Hordeum vulgare* before fertilization, and concentric ER circles were observed in the cytoplasm after fertilization. ER with expanded cisterns was found near the cell nucleus [6]. In the antipodal cells of wheat at the mature megagametophyte stage, both parallel cisterns and concentric circles covering the areas of the cytoplasm with organelles were observed [19].

At various stages of the development of antipodal wheat cells, we observed three types of reticulum structure. At the early stage of differentiation, a network of extended flat (10 nm) ER cisternae (≥10 microns in length) occurred and, at the same time, short tubes (diameter, 40 nm) were detected. At the middle and late stages of differentiation, concentric circles (up to 5 microns in diameter) were observed.

It is known that plants accumulate and store proteins in specialized protein storage vacuoles (PSV) and multivesicular bodies (MVBs), which are necessary for the development and maturation of seeds. These stored proteins are later mobilized to provide nutrients to germinating seeds. During the germination of bean seeds (*Vigna radiata*), vacuolar sorting receptor (VSR) proteins and hydrolytic enzymes are synthesized de novo. Using immunoelectronic microscopy, it was possible to identify the cysteine protease aleurain, and show that VSR and aleurain are colocalized in MVBs and in the PSV of germinating seeds. Thus, MVBs in germinating seeds perform a dual function: as a storage for proteases that are physically separated from the PSV in mature seed and as an intermediate for VSR receptor-mediated delivery of proteases from the Golgi apparatus to the PSV for protein degradation during seed germination [41].

We observed MBTs located in the cytoplasm, in exocytotic vacuoles associated with the plasma membrane, in the apoplast, and between rare disordered hemicellulose fibrils of the cell walls of antipodal cells during programmed cell death (PCD). We assume that these multivesicular bodies perform a similar function of storing proteins and simultaneously transporting newly synthesized proteases to the endosperm for subsequent degradation during seed germination.

The key characteristics of the antipodal cell nuclei and cytoplasm are summarized in Table 5 and Table 6.

Previously, we assumed that the events observed in the antipodal cells of fertilized wheat embryo sacs such as the release of cytochrome c, DNA fragmentation, and chromatin compaction were similar to the events that occur during apoptotic cell death according to the classification of Reape and McCabe [42] or during programmed necrosis according to the van Doorn classification [43]. Nevertheless, wheat antipodal cell death has its own characteristic features, which include a series of complex transformations of nuclei, the segregation of the nucleolar material, and the extrusion of the components of nucleolus and chromatin into the endosperm [3].

The identification of key transcripts and the elucidation of the gene expression profiles in antipodal cells are important to understand the function of antipodal cells. Gene expression data in antipodal wheat cells are not available in the literature, so any progress in this area is significant. Ta54227 was used as a reference gene. Genes expressed in antipodal *Arabidopsis* cells have been identified [44]. Of these, we selected genes that had orthologs in wheat and a known function. One of the possible functions of antipodal cells is protective function, so we selected such genes for the present study including cytochrome p450, which is expressed in *Arabidopsis* antipodal cells, and the Hsp70 heat shock gene, which is expressed in cells with polytene *Diptera* chromosomes. In the antipodal cells of wheat, these genes are also highly expressed. The expression of the hydrolase gene (glycosyl hydrolase family 17 protein) and the gene associated with steroid metabolism (oxysterol-binding family protein), actively expressed in antipodal *Arabidopsis* cells, was also studied. Because plant hormones regulate virtually all processes during plant development, we selected a gene associated with ethylene metabolism that is expressed in *Arabidopsis* antipodal cells and a gene associated with auxin metabolism because it has been shown that auxin affects the initial stages of the ontogenesis of antipodal cells in *Zea mays* [45].

The higher expression of hydrolase gene and gene associated with steroid metabolism in antipodal wheat cells in comparison with the whole embryo sac can be explained by the intensive metabolic function of antipodal cells. The high expression of cytochrome p450 and Hsp70 genes can reveal the protective role of antipodal cells. The increased expression of a gene associated with ethylene metabolism can indicate its role in the development of antipodal cells, and the lower expression of a gene associated with auxin metabolism can indicate that auxin takes part only in the initial stages of the ontogenesis of antipodal cells during proliferation, but not during the process of their differentiation.

The increased expression of the cytochrome p450 gene in roots can be explained by the fact that the root is a plant organ where the maximum detoxification and the conversion of many insoluble compounds into soluble ones occurs. It is clear that the level of work of the cytochrome p450 gene of antipodal cells was noticeably lower. Since these cells are most likely designed to create matrices of proteins necessary for the endosperm and to provide protection from biotic and abiotic stresses, an increase in the expression of genes encoding Hsp70 was detected in the antipodal cells in comparison with the whole embryo sac, and even in comparison with the root cells.

In further studies, it would be of interest to identify more tissue-specific genes and examine the gene expression profiles associated with antipodal wheat cells within a complete transcriptome. This is a technically difficult task, which has not yet been achieved because obtaining samples of antipodal cells in sufficient quantity and adequate purity is difficult.

Antipodal cells act as a barrier for trophic tissue in the embryo sac, located between maternal tissues and the tissues formed during double fertilization. Most studies [1,4,6,7,8,10,12,13,14,15,16,17,18,19,20,21,22,23] suggest a secretory and feeding function of the antipodal cells of the female gametophyte. This is supported by the presence of polytene chromosomes, large vacuolated nucleoli, numerous cytoplasmic organelles, and a well-developed synthetic ER and Golgi apparatus. It is believed that accumulated nutrients are transferred to the developing nuclear endosperm [1,15,17,18]. In addition, antipodal cells can secrete active substances (possibly hormones) necessary for the development of the endosperm [1,6]. Brink and Copper considered antipodal cells as the most prominent and most active tissue in the embryo sac [4]. Engell considered them as the metabolic center for the absorption and production of nutrients [6].

In recent studies [1,3], the phenomenon of extruding chromosome sections and nucleolar components into the vacuoles of the adjacent syncytium of the endosperm has been demonstrated. During the study of the ultrastructure of antipodal cells, we found the extrusion of nucleolar material from the nucleus into the cytoplasm. The transition of cytoplasmic components and organelles through wide channels of the cell wall was demonstrated during the death of the wheat antipodal cells [20]. We also observed these phenomena in dying wheat antipodal cells. These rare types of secretion indicate a significant role for products produced by antipodal cells to ensure the normal development of the endosperm. Perhaps they produce both nutrients and numerous antistress factors that maintain the development of the endosperm of the grain, which, in turn, further ensures the normal development of the embryo.

After double fertilization, the development of antipodal cells is directly associated with the ontogenesis of the endosperm. During their differentiation, antipodal cells produce the products necessary for the proper development of the endosperm. Following completion, the endosperm induces their death [1,3]. Thus, it is of interest to study these relationships in seeds with developmental anomalies. In rye–wheat and rye–barley hybrids, the volume of antipodal cell complexes is smaller compared to that of the original species. A decrease in the number of antipodal cells in the complex, a decrease in the size of the antipodal cells, or a decrease in their functional activity causes the endosperm to not fully develop, and the so-called “unfulfilled grain” occurs [4,5]. The disruption of the structure of the mature grain confirms the importance and necessity of the products produced by the antipodal cells for the proper development of the endosperm. There is an opinion that *Secale* sperms during double fertilization and the formation of hybrids somehow affect the ability of antipodal cells to synthesize substances [4]. If the cells of the antipodal complexes of wheat, *Triticale*, and rye decrease the size of the antipodal cell nuclei before fertilization, the formation of normal grain is disrupted. Therefore, the estimation of the volume of antipodal nuclei was proposed as a marker for the future yield of these plants. This is more evidence of the importance of the antipodal cells to cultivated cereals [46].

When antipodal cells are differentiating and actively functioning, the death of nucellus cells occurs. Some studies have suggested that antipodal cells participate in both the secretion of hydrolytic enzymes that destroy nucellus cells and in the selective absorption and transport of substances from the nucellus tissue to the endosperm [6,21]. We also observed the phenomena of PCD of the nucellus cells during the differentiation stages of the antipodal wheat complex. Morphologically, cell death in nucellus cells resembles vacuolar cell death. The participation of antipodal cells in the cell death process of the nucellus appears to be relevant.

In some studies, the idea that antipodal cells nourish the embryo was expressed, but this was criticized because, during the period of maximum synthetic and secretory activity of antipodal cells in the embryo, neither intensive divisions nor differentiation processes occur. They begin only after the complete degradation of the antipodal cells [4].

The formation of complexes of highly polyploid antipodal cells with giant nonclassical polytene chromosomes in the embryo sacs of all cultivated cereals is a typical example of the functional somatic polyploidization of genomes. Giant polytene chromosomes transcribe protein that ensures the proper formation and protection of the endosperm, the cells of which all substances necessary for the development of the embryo during seed germination accumulate.

## 5. Conclusions

We have traced the ontogenesis of wheat antipodal cells including differentiation and programmed cell death. Of particular interest is the stage of antipodal differentiation, when polytene chromosomes are formed and the maximum gene expression necessary for the existence and protection of the emerging nuclear endosperm occurs. For the first time, all changes of the nuclear domains—the nuclear envelope, polytene chromosomes, and the nucleolus—are clearly shown. The dynamics of all cytoplasmic organelle structures involved in synthetic processes has been shown. These changes reflect the possible functions and the synthesis of new products specific to a particular stage of development. For the first time, we were able to detect an increase in gene expression, the products of which can protect the developing endosperm coenocyte. We do not give up hope that we will be able to significantly expand the list of these genes in the near future. The process of the wheat antipodal cells’ PCD morphologically is a kind of apoptosis-like variant PCD, specific to highly polypoid cells with giant polytene chromosomes.

The choice of isolated embryo sacs with the antipodal cells of cultivated cereals as a model allows us to solve not only fundamental questions of cell biology (the structure of polytene chromosomes, PCD mechanisms, plant cell ontogenesis), but also issues of increasing yields, because antipodal cells directly provide grain formation.

## Figures and Tables

**Table 1:** The genes and their orthologs studied in *Triticum*.

*Arabidopsis* Gene	Function	Assumed Ortholog in *Triticum*
*YUCCA 9*	Auxin metabolism	*TraesCS2A02G011500*
*AT3G12580*	Cytochrome p450	*TraesCS7D01G427500LC.1*
*AT5G01860*	C_2_H_2_ and C_2_HC zinc finger superfamily protein	*TraesCS7A02G276700*
*At1g77790*	Glycosyl hydrolase family 17 protein	*TraesCS1A02G312700*
*At3g14630*	Hsp70	*LOC543123*
*At2g31030*	Oxysterol-binding family protein	*TraesCS4B02G042300*

**Table 2:** The gene expression levels in the antipodal cells.

Antipodal Cells					
*Ta54227*	1	1	1	Mean	Standard Derivation
*YUCCA 9*–auxin metabolism	0.218	0.157	0.145	0.173	0.039
Cytochrome p450	4.8	4.1	4.68	4.527	0.374
C_2_H_2_ and C_2_HC zinc finger superfamily protein	0.913	0.923	0.866	0.901	0.030
Glycosyl hydrolase family 17 protein	0.703	0.498	0.55	0.584	0.107
Hsp70	11	10.4	9.7	10.367	0.651
Oxysterol-binding family protein	2	1.4	1.3	1.567	0.379

**Table 3:** The gene expression levels in the cells of the embryo sac.

Embryo Sac					
*Ta54227*	1	1	1	Mean	Standard Derivation
*YUCCA 9*–auxin metabolism	0.333	0.312	0.234	0.293	0.052
Cytochrome p450	2	2.7	2.4	2.367	0.351
C_2_H_2_ and C_2_HC zinc finger superfamily protein	0.523	0.463	0.324	0.437	0.102
Glycosyl hydrolase family 17 protein	0.168	0.152	0.124	0.148	0.022
Hsp70	1.1	1.2	0.967	1.089	0.117
Oxysterol-binding family protein	0.287	0.214	0.241	0.247	0.037

**Table 4:** The gene expression levels in the root cells.

Root Cells					
*Ta54227*	1	1	1	Mean	Standard Derivation
*YUCCA 9*–auxin metabolism	1.7	1.8	1.4	1.633	0.208
Cytochrome p450	38.7	30.8	63	44.167	16.782
C_2_H_2_ and C_2_HC zinc finger superfamily protein	4.6	4.2	4.1	4.300	0.265
Glycosyl hydrolase family 17 protein	0.384	0.591	0.52	0.498	0.105
Hsp70	5.1	7.5	4.4	5.667	1.626
Oxysterol-binding family protein	1.5	1.7	2.2	1.800	0.361

**Table 5:** The characteristics of the antipodal cell nuclei during differentiation and death.

	Nucleus			
Ontogenesis Stage	Form	Chromosomes	Nucleolus	Nuclear Envelope
Early differentiation 1–3 days after pollination	Round	Undifferentiated	1–4	Minor invaginations
Middle differentiation 3–4 days after pollination	Ovoid	Individual chromosomes	1–2	Deep invaginations
Late differentiation 4 days after pollination	Elongated	Individual chromosomes	1–2	Deep invaginations
Early death 4–5 days after pollination	Elongated	Compacted chromosomes with lacunae	1–2, segregated components of nucleoli	Deep extended invaginations, ruptures, extrusion of nuclei and nucleolus
Late death 6–7 days after pollination	Elongated and flattened	Converged or united chromosome territories, nuclei fragmentation	1–2, segregation, and flattening	Deep extended invaginations, ruptures, extrusion of nuclei and nucleolus

**Table 6:** The characteristics of the antipodal cell cytoplasm during differentiation and death.

	Cytoplasm						
Ontogenesis Stage	ER	Golgi Apparatus	Mitochondria	Plastids	Cytoskeleton	Vacuoles	Cell Wall
Early differentiation 1–3 days after pollination	Narrow cisternae	5–7 cisternae with expanded ampoules	Rounded (d, 0.5 microns)	Spherical, oval, rod-shaped, cup-shaped	Thin network	Large, from both sides of the nuclei	Uneven, exocytosis
Middle differentiation 3–4 days after pollination	Extended tanks, concentric circles	5–7 cisternae with expanded ampoules	Oval (0.5–0.8 microns), cup-shaped	Rod-shaped, cup-shaped	Thin network	Large	Exocytosis
Late differentiation 4 days after pollination	Concentric circles, short tubes	5–7 cisternae with expanded ampoules	Oval (0.5–0.8 microns), cup-shaped	Rod-shaped, cup-shaped	Thin network	Large	Exocytosis
Early death 4–5 days after pollination	Expanded cisternae	5–7 cisternae with small ampoules	Oval (0.5–0.8 microns), cup-shaped	Rod-shaped	Strands between invaginations of the nucleus	Large	Exocytosis
Late death 6–7 days after pollination	Expanded cisternae	5–7 curved cisternae, closed in a ring	Oval (0.5–0.8 microns), cup-shaped	Rod-shaped	Rare	Large	Exocytosis

## Data Availability

Not applicable.

## Supplementary Materials

The following supporting information can be downloaded at: https://www.mdpi.com/article/10.3390/biology11091340/s1. Table S1: Primers.
