# Supplementary material for: Wheat Antipodal Cells with Polytene Chromosomes in the Embryo Sac Are Key to Understanding the Formation of Grain in Cereals
Source: Biology (Basel). 2022 Sep 11;11(9):1340. doi: 10.3390/biology11091340 (PMC9495325; doi:10.3390/biology11091340)
Supplement: Supplementary file 1 [file biology-11-01340-s001.zip › biology-1875414-supplementary.pdf]

Table S1. Primers.

Gene	Function	Primers, Seq 5′ to 3′
LOC543123F	Hsp70	TGAACCCCATCAACACCGTC
LOC543123R	Hsp70	TTAATGTCACTCTGACAGGGCA
TraesCS7D01G427500LC.1F	Cytochrome p450	CGTGATCGCCGTGATTCTTC
TraesCS7D01G427500LC.1R	Cytochrome p450	TCGAGGCTCTTGAGGACCATA
TraesCS1A02G312700F	Glycosyl hydrolase family 17 protein	GGCGTACAACAACAACGTGA
TraesCS1A02G312700R	Glycosyl hydrolase family 17 protein	GAACAGGTACGTCTCCACGG
TraesCS2A02G011500F	Auxin metabolism	TCATGGGCAAGTCCACCTTC
TraesCS2A02G011500R	Auxin metabolism	TCTTGTCGACGAACCAGAGC
TraesCS4B02G042300F	Oxysterol-binding family protein	GCTGTAAGCCCTTCAACCCT
TraesCS4B02G042300R	Oxysterol-binding family protein	AGCCATTTCCCTCACAGTGG
TraesCS7A02G276700F	C_2H_2 and C_2HC zinc finger superfamily protein	GGAGATAGTGGCCAAGCTGC
TraesCS7A02G276700R	C_2H_2 and C_2HC zinc finger superfamily protein	CGTGCACACGATGAACGTAG
Ta54227F	Reference	CAAATACGCCATCAGGGAGAACATC
Ta54227R	Reference	CGCTGCCGAAACCACGAGAC
